# Supplementary material for: Regulatory Patterns of Crp on Monensin Biosynthesis in Streptomyces cinnamonensis
Source: Microorganisms. 2020 Feb 17;8(2):271. doi: 10.3390/microorganisms8020271 (PMC7074812; doi:10.3390/microorganisms8020271)
Supplement: Supplementary file 1 [file microorganisms-08-00271-s001.pdf]

Supplementary Material

Regulatory patterns of Crp on monensin biosynthesis in *Streptomyces cinnamonensis*

Chun-Yan Lin [a,1], Yue Zhang [a,c,1], Ji-Hua Wu [a], Rong-Hui Xie [a], Jianjun Qiao [a,b] and Guang-Rong Zhao [a,b,*]

[a] Frontier Science Center for Synthetic Biology and Key Laboratory of Systems Bioengineering (Ministry of Education), School of Chemical Engineering and Technology, Tianjin University, Yaguan Road 135, Jinnan District, Tianjin 300350, China; lcy87828@126.com (C-Y.L.) ; zhangy@tib.cas.cn (Y.Z.); 18846321904@163.com (J-H.W); 1835327730@qq.com (R-H.X); jianjunq@tju.edu.cn (J.Q)

[b] SynBio Research Platform, Collaborative, Innovation Center of Chemical Science and Engineering (Tianjin), Tianjin 300072, China

[c] Present address: Tianjin Institute of Industrial Biotechnology, Chinese Academy of Sciences, Tianjin 300308, China

[1] C-Y.L. and Y.Z. contributed equally to this work.

* Correspondence: grzhao@tju.edu.cn (G-R. Z.); Tel.: +86-22-85356580; Fax: +86-22-27403389

Table S1 Primers used in this study

Primer name	DNA sequences (5'–3')
**For construction of *crp* overexpression and antisense RNA plasmids**	
CZ-F	GCTCTAGAGTGGACGACGTTCTGCGG
CZ-R	TTTTCCTTTTGCGGCCGCTCAGCGCGACCGCTTGGC
CS1-F	CCCAAGCTTCAGTGGTGGTGGTGGTGGTGCAGATCT CGCGGCCTGCTCGTCATC
CS1-R	TGCTCTAGACAGTGGTGGTGGTGGTGGTGCATGCAT ACGGACCCCTGATATGCG
**For semi-quantitative RT-PCR**	
16S-F	CTGCCCTTCACTCTGGGACA
16S-R	TCCGACTTCATGGGGTCGAG
acs-F	TCTGGGCCGAGCAGGCCCGC
acs-R	GCTGACGGCCTCGTCCACGG
pckA-F	ATGTACGTCGTGCCCTTCTG
pckA-R	TCTCGACGGTCCAGCCGGAG
accB-F	ACTCGGGCGGCGCCCGCATC
accB-R	ACGTGTTCGATGACGCCGTG
ccr-F	GTCAACTACAACTCCGTGTG
ccr-R	AACTGCGTGGCGTACGACCC
mutB-F	ACGAGCGCTACAAGATGATC
mutB-R	CCCGAAGCCGTCCGCGAGGG
epi-F	ATGCTGACGCGAATCGACCA
epi-R	TCAGTGCTCCGGTGCCTCGG
acdH-F	TGCGCACGGAGGTGCTGCCG
acdH-R	GCAGGGACAGGCCTGTGGTG
atoB-F	TGCCAGTGCGGCTCCTCGCA
atoB-R	ATGAGGGCGGCCGCGCCGTC

TU1-F	GACGGCAATCTGCACTACGG
TU1-R	TGATGTCGGTCAGTTCGAGGAC
TU2-F	ATGTGGGAGAAGCGGGTGG
TU2-R	GGTACGGATGGACTGGTTGACG
TU3-F	GCTCGGGACTGCGGAAGAA
TU3-R	CGTGCGACGGGAAGCCATT
TU4-F	TGGAGCGGTGTTTCCCTGTG
TU4-R	AGCCTCCCGTCGTGCTACCT
TU5-F	TGGAACGGCGTCTCCCTGTG
TU5-R	CGTGCGATGTGGCTGTGGTCT
TU6-F	CCCACGACTACCTGACGCTGAT
TU6-R	ACGCTGCCCTGTTCCCACTG
TU7-F	TGACGAGCAGGATGAAGAAGGA
TU7-R	AACATGGACAACCCGGAACAC
TU8-F	GGGAACTCGCCGACCTCAT
TU8-R	TGCTGGTGCGTGTTCAGATAGA
monH-F	CCGCTTGGCGTCCTGGAACA
monH-R	TCTGATGGGCGTACTCGGTGAA
crp-F	GTGGACGACGTTCTGCGG
crp-R	TCAGCGCGACCGCTTGGC

Table S2 Putative gene functions of 57 DEGs

Glycolysis (10):

Gene	Function
Scin6520	Aldose 1-epimerase
Scin0389	Acetyl-CoA synthetase
Scin4931	Phosphoenolpyruvate carboxykinase
Scin0555	Probable aldehyde dehydrogenase
Scin1986	NAD-dependent alcohol dehydrogenase
Scin3280	Alcohol dehydrogenase
Scin4853	Putative aldehyde dehydrogenase
Scin0875	Glucokinase
Scin2469	Glucosyl-3-phosphoglycerate phosphatase
Scin4003	3-methyl-2-oxobutanoate dehydrogenase subunit alpha

Fatty acid degradation (20):

Gene	Function
Scin2471	Putative cytochrome P450
Scin3350	Cytochrome P450
Scin6039	3-ketoacyl-CoA thiolase
Scin2551	Long-chain-fatty-acid--CoA ligase
Scin1513	Acyl-CoA dehydrogenase
Scin2065	Long-chain-fatty-acid--CoA ligase
Scin1599	Glutaryl-CoA dehydrogenase
Scin0986	NAD-dependent alcohol dehydrogenase

Scin6804	Fatty acid oxidation complex subunit alpha
Scin2688	Long-chain-fatty-acid--CoA ligase
Scin2280	Alcohol dehydrogenase
Scin0382	Long-chain specific acyl-CoA dehydrogenase
Scin7342	Acyl-CoA dehydrogenase
Scin4869	Acyl-CoA dehydrogenase
Scin2743	Long-chain specific acyl-CoA dehydrogenase
Scin7189	Acetyl-CoA acetyltransferase
Scin2312	C26-monooxygenase
Scin4504	Long-chain-fatty-acid--CoA ligase
Scin6924	Putative cytochrome P450
Scin7256	C-12 hydroxylase

Carbon metabolism (27):

**Gene**	**Function**
Scin0289	Long-chain specific acyl-CoA dehydrogenase
Scin5352	Coenzyme B12-dependent methylmalonyl-CoA mutase B subunit
Scin1671	Phosphoserine phosphatase
Scin6706	Long-chain specific acyl-CoA dehydrogenase
Scin7512	Glucokinase
Scin6534	Aspartate ammonia-lyase
Scin0885	Alcohol dehydrogenase
Scin7131	Putative protein
Scin7361	Uncharacterized pyridoxal phosphate-dependent protein
Scin1818	Glucose-6-phosphate 1-dehydrogenase
Scin6197	Methylmalonyl-CoA epimerase
Scin2763	Long-chain specific acyl-CoA dehydrogenase
Scin4012	Acyl-CoA carboxylase complex B subunit
Scin5365	Phosphate acetyltransferase
Scin5364	Acetate kinase
Scin1004	Pyruvate phosphate dikinase
Scin6441	Crotonyl-CoA reductase
Scin1526	Glucosyl-3-phosphoglycerate phosphatase
Scin7132	Succinate dehydrogenase flavoprotein subunit
Scin7133	Succinate dehydrogenase iron-sulfur subunit
Scin2281	Fumarate reductase (CoM/CoB) subunit A
Scin0394	NAD-dependent malic enzyme
Scin2459	Acetate kinase
Scin2569	Acyl-CoA dehydrogenase
Scin5218	Putative siderophore biosynthesis protein
Scin5020	Fatty acid oxidation complex subunit alpha
Scin5021	Acyl-CoA dehydrogenase
